# Understanding Antenatal Care Service Quality for Malaria in Pregnancy through Supportive Supervision Data in Tanzania

**DOI:** 10.4269/ajtmh.23-0399

**Published:** 2024-02-06

**Authors:** Goodluck Elias Tesha, Stella Makwaruzi, Rachel Haws, Jadmin Mostel, Abdallah Lusasi, Samwel Lazaro, Sijenunu Mwaikambo, Frank Chacky, Erik Reaves, Chonge Kitojo, Naomi Serbantez, Gladys Tetteh, Katherine Wolf, Lolade Oseni

**Affiliations:** ^1^ U.S. President’s Malaria Initiative Impact Malaria Project, Dar es Salaam, Tanzania;; ^2^ Department of International Health, Johns Hopkins Bloomberg School of Public Health, Baltimore, Maryland;; ^3^ U.S. President’s Malaria Initiative Impact Malaria Project, Washington, District of Columbia;; ^4^ Ministry of Health, National Malaria Control Program, Dodoma, Tanzania;; ^5^ U.S. President’s Malaria Initiative, U.S. Centers for Disease Control and Prevention, Dar es Salaam, Tanzania;; ^6^ U.S. President’s Malaria Initiative, United States Agency for International Development, Dar es Salaam, Tanzania

## Abstract

Malaria in pregnancy (MiP) is associated with maternal anemia, spontaneous abortion, and infant and maternal death. In Tanzania, MiP service data are collected through routine Malaria Services and Data Quality Improvement (MSDQI) supportive supervision rounds at antenatal care (ANC) facilities. Using structured assessment tools, the U.S. President’s Malaria Initiative Impact Malaria Project reviewed two annual rounds of MSDQI data (492 facilities in 2021 and 522 facilities in 2022), including ANC records and client satisfaction interviews. We assessed coverage of key MiP care components, used logistic regression to analyze uptake of the recommended three or more doses of intermittent preventive treatment in pregnancy (IPTp3+), and assessed client satisfaction. Coverage of most MiP care components exceeded 80%; however, only 38% of women received all components. Odds of receiving IPTp3+ were much lower among late ANC initiators than among those who initiated ANC during their first trimester (odds ratio [OR], 0.46; 95% CI, 0.38–0.57). Uptake of IPTp3+ increased almost exponentially by number of ANC visits. Women with seven visits were 30 times more likely than those with three visits to receive IPTp3+ (OR, 30.71; 95% CI, 11.33–83.22). Just 54% of clients had anemia screening and only 46% received IPTp3+. Client satisfaction with services and provider communication was high (98% and 97%, respectively); only 8% of client visits exceeded 3 hours. Increased ANC visits could boost IPTp3+ coverage. Routine MSDQI supportive supervision data are useful to assess quality of care, identify service delivery gaps, and guide policies to improve quality of MiP services.

## INTRODUCTION

Malaria in pregnancy (MiP) is a major global public health problem associated with increased maternal anemia, intrauterine growth restriction, stillbirth, and infant and maternal death.^1^ *Plasmodium falciparum* malaria infections contribute to an estimated 50,000 maternal deaths and 200,000 stillbirths annually.^2^ Control of MiP in Tanzania relies on a three-pronged approach recommended by the WHO: intermittent preventive treatment in pregnancy (IPTp) with at least three doses of sulfadoxine–pyrimethamine (SP) beginning early in the second trimester and administered at least 1 month apart, prompt case management, and use of insecticide-treated nets (ITNs), which are distributed during antenatal care (ANC).^3^ In Tanzania, as part of prevention and sentinel surveillance, all women are to be screened for malaria at their first antenatal visit; those negative for malaria and eligible for IPTp receive one dose of intermittent preventive treatment in pregnancy (IPTp1) (and additional doses during subsequent visits), whereas those positive for malaria are given a course of antimalarials and begin IPTp the following visit. Tanzania’s 2021 to 2025 Malaria Strategic Plan aims to achieve 85% coverage of the recommended three or more doses of intermittent preventive treatment in pregnancy (IPTp3+) (when not contraindicated), 85% use of ITNs by pregnant women, and 100% prompt, high-quality case management by 2025.^4^

For more than 20 years, MiP interventions have been delivered through ANC platforms, with the expectation that well-integrated services will lead to improved health outcomes.^5^ High coverage of ANC, and quality MiP services delivered during these visits, are essential to prevent malaria-associated morbidity and mortality among pregnant women in countries with a high burden of malaria. However, globally, MiP service coverage provided through ANC has lagged behind other ANC care components.^6^

In Tanzania, application of quality improvement approaches has increased coverage of key MiP interventions and contributed to improved services.^7^ The Tanzania Ministry of Health (MOH) delivers MiP and other ANC services to pregnant women through an integrated platform guided by the National Malaria Control Program (NMCP) and the Reproductive and Child Health Services (RCHS) Department. To ensure quality of reproductive health and ANC services, including MiP services, the NMCP and the RCHS Department collaboratively developed a structured supervision tool called Malaria Services and Data Quality Improvement (MSDQI) to assess readiness to provide malaria services, clinical performance under observation, data completeness, adherence to guidelines, and client satisfaction.

The MSDQI tool evolved from the checklist-based Outreach Training and Supportive Supervision (OTSS) approach to supportive supervision and quality improvement developed by the U.S. President’s Malaria Initiative (PMI) in 2007.^8^ In Tanzania, OTSS was expanded to include data quality audits and client satisfaction components, and to engage other facility departments involved in malaria service provision not covered under OTSS, including ANC clinics, inpatient departments, and logistics and supply chains. This integrated quality improvement package was first used in 2017 by the United States Agency for International Development–funded Boresha Afya projects operating in 11 regions of Tanzania, and was rolled out nationally by the MOH NMCP beginning in 2017.^9^ From 2019 to 2022, the PMI Impact Malaria Project continued to support the Tanzania MOH and the President’s Office of the Regional Administration and Local Government of Tanzania to implement MSDQI in three regions: Mtwara, Lindi, and Katavi.

During MSDQI supportive supervision visits, trained supervisors—including staff from implementing partners and the MOH at national, regional, and council levels—use the standardized, checklist-based set of MSDQI modules to assess diagnostic and treatment practices of ANC providers, and laboratory, inpatient, and outpatient departments. The modules include sections on facility readiness, clinical performance (involving direct observation and register/record review), and client satisfaction, among others.^10^​ The MSDQI data are collected using a tablet-based, open-source Android application to guide supervisors in providing in-person supervision. Digitized inputs generate a standard score to monitor health facility performance over time. Facilities rated as low-performing by the MSDQI tool are provided additional MSDQI supervision and mentorship, including development and monitoring of quality improvement plans using data from the MSDQI tool and data reported in Tanzania’s national health management information system (HMIS). The Tanzania NMCP works to ensure that all facilities receive at least one MSDQI visit per year.

### Measuring quality of malaria in pregnancy services.

Quality of care can be assessed through numerous modalities, including service availability and readiness, provider practices, service provision, and client experience. Because service delivery of components in a package of care can vary widely, assessment of service quality using more than one data source can facilitate identification of gaps.^11^ Multiple national surveys have evaluated ANC quality through facility readiness and provider performance, finding some limitations in staff availability, infrastructure, and specialized commodities.^12^^,^^13^ Provider behavior, attitudes, communication, discriminatory treatment, and failure to ensure privacy can affect the quality of ANC care.^14^^,^^15^ Service provision data permit measurement of intervention coverage by indicating whether clients received care according to clinical guidelines. Several studies have used service provision data to assess care components received as a measure of “effective coverage” of ANC.^16^^–^^18^ Although some studies of ANC quality included MiP components or used integrated ANC and MiP quality scores,^6^ there is a dearth of evidence focused on receipt of specific MiP care components during ANC.

Client experiences are more subjective and multifaceted than service records. Client experiences encompass factors beyond services received, including provider communication and behavior, privacy and respect, facility conditions, wait times, costs incurred, and financial, geographic, or social difficulties accessing care.^13^^,^^19^ However, client satisfaction drives demand for and uptake of services, as satisfied clients are more likely to return and to recommend ANC services to others. In 2017, the Lancet Global Health Commission on High-Quality Health Systems called for research linking patient experience with health-care use.^20^ Satisfaction data can identify areas to improve patient experience and increase use.^19^

### Study aims.

Provision and documentation of key care components during ANC serve as a proxy for MiP service quality. Data on service provision vis-a-vis MiP guidelines and client satisfaction can be triangulated to describe quality of MiP care. We conducted a retrospective analysis of ANC register and client satisfaction data collected through MSDQI supportive supervision visits to facilities in PMI Impact Malaria Project areas in Tanzania. The descriptive analysis elucidates the quality of MiP care components provided during ANC. In this article, we describe how MiP care components are provided at supported facilities through several lenses: coverage of MiP interventions during ANC, how gestational age at first ANC visit affects timing and doses of IPTp, and client satisfaction with ANC services.

## MATERIALS AND METHODS

### Malaria Services and Data Quality Improvement setting, approach, and data sources.

Reproductive health services, including MiP services, are offered by three types of facilities providing primary health care in Tanzania: district hospitals, health centers, and dispensaries. PMI Impact Malaria supported implementation of two annual rounds of MSDQI supportive supervision (2021 and 2022) at hospitals, health centers, and dispensaries providing reproductive health services across three regions in southeastern and western Tanzania: Lindi, Mtwara, and Katavi. During each round of MSDQI supervision at each facility, trained MOH malaria supervisors at the district level conducted a retrospective review of ANC register records of 10 women who had recently given birth. Supervisors also collected primary data by surveying two current ANC clients about services they received and their satisfaction with those services on the day of supervision. During the second round (2022), a subset of facilities with inpatient services and malaria microscopy services were visited twice. Supervisors were trained in supportive supervision skills and had received orientation on the MSDQI tool, including electronic data collection. All data collected via the electronic MSDQI tool were uploaded to the HMIS.

Record reviews followed MSDQI guidelines, retrieving 10 ANC records for pregnant women who had initiated ANC care 9 months prior to the MSDQI visit. This strategy assumed that all women would have delivered by the time of the MSDQI supervision visit; each record was confirmed in the labor and delivery register. If fewer than 10 eligible records were in the register, all available records were selected. Starting from the most recent record 9 months prior to supervision and working backward, the 10 most recent records were identified. Sampling for the client satisfaction component followed MSDQI tool guidelines, selecting the first two clients attending their first ANC visit on the day of supervision. If there were no new clients, MSDQI guidelines allowed sampling of clients attending later ANC visits.

### Study design.

PMI Impact Malaria, in collaboration with regional and district health management teams, conducted a retrospective review of service provision records and responses to client satisfaction surveys collected during the two annual rounds of MSDQI supportive supervision (2021 and 2022). Facilities that received supportive supervision visits during either round were included. The retrospective analysis featured three categories of analyses using Stata version 14.2 (StataCorp, College Station, TX): 1) frequency analysis to compute coverage of key MiP interventions; 2) logistic regression analyses to assess coverage of MiP components by facility type, uptake of IPTp by facility type; and uptake of IPTp based on gestational age at first ANC visit and number of ANC visits; and 3) frequency analysis to evaluate client satisfaction with services.

### Tools and indicators.

Service provision data were extracted from ANC register records retrieved during MSDQI supervision and exported to Excel (Microsoft, Redmond, WA). Using the MSDQI tool, zeros recorded in the register by supervisors indicate a service that was not provided, so completed fields serve as a proxy of coverage. Indicators extracted included gestational age at first ANC visit, whether hemoglobin level was tested at the first ANC visit and later visits, whether malaria testing/screening occurred at the first ANC visit, whether malaria rapid diagnostic test (mRDT) results were recorded, hematinic provision (iron and/or folic acid, any dose), ITN provision, and total number of IPTp doses received.

The MSDQI tool’s client satisfaction module was structured as multiple-choice questions with two or three possible responses (i.e., yes/no, yes/partially/no, or yes/no/not applicable). In addition to client responses, the client satisfaction tool also included supervisor appraisals of client explanations of how to take dispensed medications at home and when to return to the clinic. Variables in the MSDQI client satisfaction module included whether women had received key MiP care components, whether they received prescribed medications or commodities/services and provider communication, total time spent at the facility, and satisfaction with services.

### Data analysis.

#### Service provision data.

Before analysis, we cleaned the data to remove duplicates. We assessed completeness of register data and used frequency analysis to compute coverage of MiP care components using the indicators mentioned earlier and the median number of ANC visits by trimester of ANC initiation. Using Stata statistical software (StataCorp), we also used logistic regression to compute odds ratios (ORs) for the uptake of total IPTp doses based on gestational age at first ANC visit. Each of these regressions were restricted to the subset of women receiving each total dose, with women in that subset initiating ANC at ≤12 weeks’ gestation serving as the reference group. We also conducted regression analyses to examine associations between number of ANC visits and receipt of IPTp3+, as well as between facility type and coverage of each MiP intervention and total number of IPTp doses.

Variables in the logistic regression models included binary variables for documentation of each MiP care component: month of gestation when women initiated ANC services (converted from weeks recorded in the ANC record to 4-week intervals coded as 1–10); four binary variables for receipt of a total of zero, one, two, or three or more doses of IPTp; number of ANC visits (coded as 1–9), and a categorical variable for health facility type (dispensary, health center, or hospital). To increase sample size, and because all records were unique, we combined records from the 2021 and 2022 rounds of supportive supervision, controlling for round of data collection and facility type. Gestational age categories were later reorganized into trimester variables (≤12 weeks, 13–26 weeks, and ≥27 weeks) to compute ORs.

For the IPTp uptake analyses by gestational age at the first ANC visit, we ran five separate regressions with each binary “IPTp total dose” variable as the outcome variable and month (and for three or more total doses, also trimester) of ANC initiation as the independent variable. To assess how the number of ANC visits was associated with uptake of IPTp3+, we conducted another regression analysis with the subset of women who received three or more doses as the outcome variable, number of ANC visits as the independent variable, and three visits as the reference group (the minimum to receive IPTp3). Regressions for each MiP intervention by facility type were conducted for all records where facility type was specified, with dispensaries as the reference group. Last, to assess where women receive IPTp doses, we ran three regression analyses on subsets of women who received one, two, or three or more total doses, with facility type as the independent variable, total number of doses as the outcome variable, and dispensaries as the reference group. None of the regressions compared subsets of women who received different total numbers of IPTp doses.

#### Client satisfaction data.

We used frequency analysis to assess whether key services had been provided (content of care), whether clients could explain correctly how to take medications and when to return to the clinic (effectiveness of provider communication), and client satisfaction with services. Partial satisfaction (somewhat) was analyzed separately from complete satisfaction (yes) responses.

## RESULTS

A total of 492 of 690 facilities in 20 districts in the three PMI-supported regions received supportive supervision visits during round 1 of MSDQI supervision (2021); 522 of 690 facilities received visits in round 2 (2022). Of the 562 discrete facilities visited at least once during the two rounds and included in the analysis, most (*n =* 452) were visited twice (Table 1). Most facilities were dispensaries (*n =* 466, 83%), followed by health centers (*n =* 71, 13%) and then hospitals (*n =* 23, 4%). The service provision analysis reviewed ANC records for 11,296 pregnancies (*n =* 5,079 in round 1, *n =* 6,217 in round 2) (Table 1), with 80% at the dispensary level. The proportion of clients initiating ANC in the first, second, and third trimesters, respectively, was 37% (*n =* 3,203), 58% (*n =* 5,054), and 5% (*n *= 442). Clients initiating ANC in the first, second, and third trimesters attended a median of four, three, and two visits, respectively (Table 2).

**Table 1:** Number of facilities receiving Malaria Services and Data Quality Improvement supervision

Facility type	No. of facilities in study regions, *n*	Round 1, 2021; *n *(%)	Round 2, 2022; *n *(%)	*n* (%)*
Dispensary	582	415 (84.3)	439 (84.1)	466 (82.9)
Health center	70	61 (13.5)	65 (12.5)	71 (12.6)
Hospital	38	14 (2.8)	16 (3.0)	23 (4.1)
Unspecified	–	2 (0.4)	2 (0.4)	2 (0.4)
Total	690	492	522	562

* Most facilities were visited in both rounds.

**Table 2:** Number of antenatal care records reviewed by type of facility

Facility type	No. of facilities in study regions, *n*	Round 1, 2021; *n *(%)	Round 2, 2022; *n *(%)	*n* (%)*
Dispensary	582	4,309 (84.8)	4,694 (75.5)	9,003 (79.7)
Health center	70	610 (12.0)	1,224 (19.7)	1,834 (16.2)
Hospital	38	140 (2.8)	279 (4.5)	419 (3.7)
Unspecified	–	20 (0.3)	20 (0.3)	40 (0.4)
Total	690	5,079	6,217	11,296

* Most facilities were visited in both rounds.

For the client satisfaction analysis, 1,035 responses (*n =* 469 in round 1, *n =* 566 in round 2) were included (Supplemental Table 1). In round 2, because facilities with inpatient and microscopy services were visited twice, 31 facilities had three or four clients surveyed; almost half the facilities (46% in round 1 and 44% in round 2) had no clients on the date of supervision.

### Coverage of key malaria in pregnancy care components.

**Figure 1.**
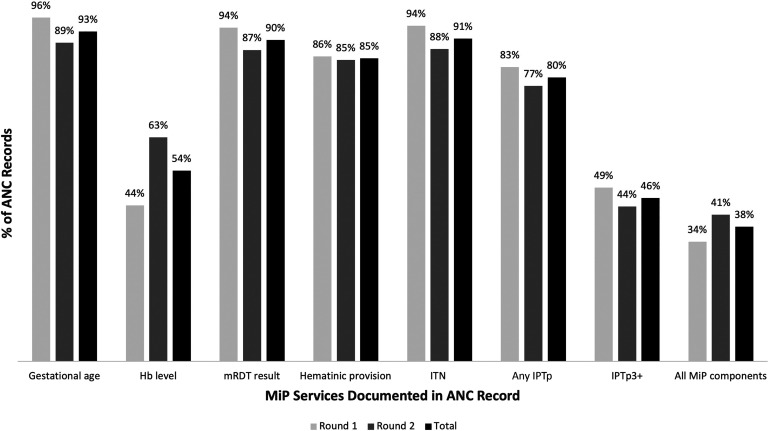
Coverage of MiP care components (ANC register review). Round 1, 492 facilities; round 2, 522 facilities; total, 562 discrete facilities. ANC = antenatal care; Hb = hemoglobin; IPTp = intermittent preventive treatment in pregnancy; Any IPTp = one or more doses of intermittent preventive treatment in pregnancy; IPTp3+ three or more doses of intermittent preventive treatment in pregnancy; ITN = insecticide-treated net; MiP = malaria in pregnancy; mRDT = malaria rapid diagnostic test.

Documented coverage of each of the MiP care components in reviewed ANC records was generally high. For gestational age and each MiP care component, the percentage of records documenting these was also consistent in both rounds, with the exception of a 19-percentage point difference in hemoglobin testing between rounds (44% versus 63% in rounds 1 and 2, respectively). Hemoglobin testing results were the least frequently recorded of all services (54% of records). Receipt of each of the other MiP care components during ANC was recorded in more than 75% of records, with the exception of IPTp3+ (Figure 1). Although 80% of records documented receipt of at least IPTp1, only 46% documented IPTp3+. Thirty-eight percent of records documented receipt of all MiP care components captured in the register (hemoglobin level, mRDT, hematinic provision, ITN provision, and any IPTp).

### Relationship between initiation of antenatal care and receipt of three or more doses of intermittent preventive treatment in pregnancy.

**Figure 2.**
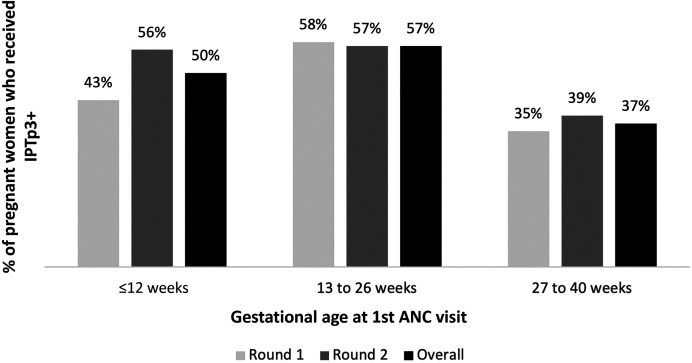
Uptake of three or more doses of intermittent preventive treatment in pregnancy (IPTp3+) by gestational age at first antenatal care (ANC) visit (frequency analysis). Round 1, *n* = 4,031; round 2, *n* = 4,711.

When they initiated ANC, most pregnant women who ultimately received IPTp3+ were in their first or second trimester of pregnancy (Figure 2). A slightly greater proportion of women who initiated ANC in the second trimester versus the first trimester received IPTp3+ (57% versus 50%, respectively, *P* <0.001). Regression analysis revealed that women who initiated ANC in the third trimester versus the first trimester were less than half as likely to receive IPTp3+ (OR, 0.46; 95% CI, 0.38–0.57) (Table 3). Women who initiated ANC during the second trimester versus the first trimester were slightly more likely to receive IPTp3+ (OR, 1.21; 95% CI, 1.10–1.32). Among women who initiated ANC after the fifth month of pregnancy, the odds of receiving IPTp3+ decreased steadily for each month ANC initiation was delayed until delivery (Table 3). There were 2,291 women (20.3%) who received zero doses of IPTp; these women were less likely to initiate ANC during the second trimester (when IPTp1 is first indicated) than the first or third trimesters.

**Table 3:** Association of gestational age at first antenatal care visit with receipt of IPTp (regression results)

No. of IPTp doses received	Gestational age at first antenatal care visit, OR (95% CI)							
	≤12 Weeks (*n* = 4,061)	Month 4 (*n* = 2,343)	Month 5 (*n* = 1,952)	Month 6 (*n* = 1,156)	Month 7 (n = 541)	Month 8 (*n* = 172)	Month 9+ (*n* = 37)	Month 10+ (*n* = 6)
0	Ref.	**0.656 (0.55–0.79), *P =* 0.00**	**0.708 (0.58–0.86), *P =* 0.00**	**0.765 (0.61–0.96), *P =* 0.02**	0.785 (0.56–1.08), *P =* 0.14	1.238 (0.78–1.96), *P =* 0.36	1.319 (0.51–3.40), *P =* 0.57	1.689 (0.20–14.49), *P =* 0.63
1	Ref.	**0.86 (0.75–0.98), *P =* 0.02**	**0.74 (0.64–0.86), *P =* 0.00**	0.92 (0.78–1.09), *P =* 0.34	**1.33 (1.07–1.64), *P =* 0.009**	**2.05 (1.47–2.88), *P =* 0.00**	**2.32 (1.17–4.63), *P =* 0.02**	2.11 (0.35–12.67), *P =* 0.41
2	Ref.	**0.79 (0.69–0.91), *P =* 0.001**	**0.85 (0.73–0.98), *P =* 0.03**	1.10 (0.93–1.30), *P =* 0.28	**1.62 (1.31–2.0), *P =* 0.00**	1.42 (0.98–2.06), *P =* 0.06	**2.28 (1.12–4.64), *P =* 0.02**	2.76 (0.46–16.58), *P =* 0.27
≥3	Ref.	**1.294 (1.16–1.45), *P* <0.001**	**1.372 (1.22–1.55), *P* <0.001**	0.99 (0.86–1.14), *P =* 0.94	**0.56 (0.46–0.68), *P =* 0.00**	**0.41 (0.29–0.58), *P =* 0.00**	**0.23 (0.10–0.50), *P =* 0.00**	0.19 (0.02–1.72), *P =* 0.14

	Timing of first antenatal care visit, OR (95% CI)		
	≤12 Weeks (*n* = 1,835)	13–26 Weeks (*n* = 3,152)	27–40 Weeks (*n* = 165)*
≥3	Ref.	**1.21 (1.10–1.32)**, ***P* = 0.00**	**0.46 (0.38–0.57), *P *= 0.00**

This table presents five separate regression analyses (by month for zero, one, and two total doses, and by month and trimester for three or more total doses). IPTp = intermittent preventive treatment in pregnancy; IPTp3+ = three or more doses of intermittent preventive treatment in pregnancy; OR = odds ratio; Ref. = reference value. Values in bold type are statistically significant.

* Third trimester.

### Relationship between number of antenatal care visits and receipt of three or more doses of intermittent preventive treatment in pregnancy.

Controlling for round number and type of facility, the likelihood of receiving IPTp3+ increased steadily with each ANC visit after reaching the minimum of three visits (Table 4). Pregnant women who completed four visits were more than four and a half times more likely to receive IPTp3+ compared with those who completed three visits (OR, 4.52; 95% CI, 3.88–5.26). Women who completed seven visits were 30 times more likely to have received IPTp3+ than those with three visits; the results describe an almost exponential relationship between number of ANC visits and IPTp3+ coverage through the eighth ANC visit. Very few women completed eight or nine visits; ORs either could not be computed or were deemed outliers.

**Table 4:** Association of number of antenatal care visits with receipt of three or more doses of IPTp (regression results)

	No. of antenatal care visits during pregnancy, OR (95% CI)						
	3	4 (*n* = 1,735)	5 (*n* = 1,161)	6 (*n* = 586)	7 (*n* = 153)	8+ (*n* = 33)	9+ (*n* = 8)
Odds of receipt of IPTp3+	Ref.*	**4.52 (3.88–5.26), *P =* 0.00**	**7.78 (6.31–9.60), *P =* 0.00**	**14.18 (9.87–20.37), *P =* 0.00**	**30.71 (11.33–83.22), *P =* 0.00**	**6.56 (2.32–18.60), *P =* 0.00**	NA^†^

IPTp = intermittent preventive treatment in pregnancy; IPTp3+ = three or more doses of intermittent preventive treatment in pregnancy; NA = not applicable; OR = odds ratio; Ref. = reference value. Regression performed on subset of women documented as receiving 3 or more doses of IPTp. Values in bold type are statistically significant.

* Three visits selected as reference based on logic that a minimum of 3 visits is required to receive at least 3 doses of IPTp.

^†^ Could not be computed because of small sample size.

### Coverage of malaria in pregnancy care components by facility type.

Provision of ITNs and gestational age documentation were significantly more likely at dispensaries than health centers or hospitals; anemia testing was more likely at health centers and hospitals than dispensaries (Supplemental Table 2). When controlling for round, regression analysis revealed that the odds of women receiving just one dose of IPTp was marginally more likely at health centers than at dispensaries (OR, 1.16; 95% CI, 1.02–1.33) (Supplemental Table 3). However, the odds of receiving IPTp3+ were somewhat greater among women seeking ANC from hospitals than dispensaries (OR, 1.34; 95% CI, 1.08–1.67).

### Client satisfaction.

Client satisfaction was comparable in both rounds. Overall, clients reported high rates of malaria testing at the first visit (98%), and receipt of tests and medicines their provider ordered (89% and 72%, respectively) (Table 5). In terms of quality and content of provider communication, most clients reported their provider had explained how to take medicines received, how to use an ITN, and when to return for their next ANC visit. Ninety-eight percent reported satisfaction with the services they received, and 97% reported satisfaction with provider communication. Ninety-two percent reported spending less than 3 hours at a visit; 40% reported visits of less than 1 hour—the Tanzania MOH ideal.

**Table 5:** Malaria Services and Data Quality Improvement client satisfaction survey results

Client response/supervisor appraisal	Round 1, 2021 (*n* = 469), %	Round 2, 2022 (*n* = 566), %	Overall (*n* = 1,035), %
Received all tests ordered by the health-care provider at this facility	88	90	89
Told by health-care provider how to take the medicines received			
Yes	86	87	86
Somewhat	8	9	9
Client could explain correctly the use of dispensed drugs at home*			
Yes	81	85	83
Somewhat	12	13	12
Told how to use an ITN	84	88	86
Told by health-care provider when to return to the health facility	96	96	96
Client could explain correctly when to return to the health facility*	94	95	95
Wait time to get all RCH services			
<1 hour	44	37	40
1–3 hours	49	54	52
>3 hours	7	8	8
Satisfied with services provided by health facility staff			
Yes	87	89	89
Somewhat	9	9	9
Satisfied with communication from health providers			
Yes	89	90	90
Somewhat	7	7	7
Tested for malaria during the first antenatal care visit^†^	96 (*n =* 296)	99 (*n =* 357)	98 (*n =* 653)

ITN = insecticide-treated net; RCH = reproductive and child health.

* Response assessed by supervisor during Malaria Service and Data Quality Improvement supervision.

^†^ Subset of clients attending first antenatal care visit.

## DISCUSSION

### Coverage as a quality indicator.

Routine data collected via the checklist-based MSDQI tool proved useful for generating a picture of how a suite of MiP services was being provided through integrated ANC in several regions in Tanzania. Overall coverage of key MiP services was high, as was client satisfaction with services; however, analysis of MSDQI data highlighted some gaps in quality of care.

Receipt of MiP services is one critical dimension of quality of care.^21^ In our study, provision of MiP care components serves as an indicator of high-quality care. Overall, although coverage of IPTp3+ was only 46%, falling far short of Tanzania’s target of 85%, the average coverage in countries implementing this strategy is 35%, demonstrating that Tanzania is performing comparatively well.^22^ In addition, coverage of most key MiP interventions during ANC was high, indicating that most facilities under MSDQI supervision in this analysis were offering quality MiP care through integrated ANC in accordance with national guidelines. Triangulated with self-reported client satisfaction, which was high across all metrics, and indications that clients understood provider communication, these results illustrate how data collected through routine supportive supervision can be used to assess quality of MiP services.

Client satisfaction with ANC is generally fairly high across studies in low- and middle-income countries, even where ANC coverage is low. In studies that have documented low satisfaction,^23^^–^^26^ high costs, disrespectful interactions with health-care providers, and long wait times are frequent complaints from clients about the quality of services received. Several studies and a systematic review and meta-analysis^26^^–^^28^ found that client satisfaction is greatest when wait times are less than 1 hour. Antenatal care clients in Mozambique reported more positive experiences and increased numbers of visits after an appointment scheduling pilot study reduced wait times, suggesting that improving client experiences may improve uptake of critical services, including IPTp3+.^29^ High client satisfaction in our study may be correlated with free services and generally acceptable wait times in the study area. Although we have no data on client perceptions of how they were treated, most reported satisfaction with provider communication. However, a multicountry meta-analysis found that the first ANC visit was associated with greater client satisfaction^30^; by targeting new ANC clients, MSDQI may overestimate client satisfaction.

### Policy and program utility of Malaria Services and Data Quality Improvement data.

At 46%, IPTp3+ uptake remains far below Tanzania’s national target of 85%, but it is in line with other studies from Tanzania reporting suboptimal uptake of IPTp3+.^31^^,^^32^ Both demand- and supply-side issues likely contribute to low uptake of IPTp. From the demand side, almost half of women presented for ANC during the second trimester, which is the ideal window during pregnancy to deliver MiP interventions. Early ANC initiators were slightly less likely than those who initiated ANC during the second trimester to receive IPTp3+, despite a greater median number of ANC visits. This may be attributable in part to IPTp guidelines, because SP is not indicated during the first trimester and it is possible that those who initiate ANC during the first trimester have several visits before they are eligible for IPTp. Late ANC initiation reduced the likelihood of receiving IPTp3+ significantly, similar to findings from Malawi, where women who initiated ANC during the first two trimesters were more than twice as likely to receive IPTp3+ than those initiating ANC during the third trimester. Although the share of late ANC initiators made up only 7.4% of our sample, this group attended a median of only two visits, in line with other studies^33^^,^^34^ that demonstrated that delaying ANC limits the total number of visits (and SP doses) possible before delivery. Late ANC initiators are more likely to be less well educated, be of greater parity, live farther from the facility, have a mistimed or unplanned pregnancy, be unable to afford opportunity costs of ANC attendance, lack partner support, or lack understanding of the benefits of ANC.^35^^–^^44^ A study of ANC satisfaction in Nigeria found that most respondents (89%) had a poor understanding of MiP services.^23^ Similar findings from central Tanzania suggest that efforts to increase demand through education about the content, timing, and purpose of MiP services during ANC could improve IPTp coverage.^42^ Coverage of IPTp3+ among early ANC adopters improved during the second round, and is potentially attributable to MSDQI (although coverage of other interventions was relatively stable between rounds).^45^ Uptake of IPTp in most countries is low even where ANC coverage is relatively high, and where governments have endeavored to solve SP stock-outs.^46^

From the supply side, facility readiness is crucial: analysis of data from the Tanzania Service Provision Assessment (SPA) found that women who attended ANC at facilities with high readiness scores were 2.1 times more likely to receive IPTp than those attending facilities with low readiness scores.^31^ Stock-outs of SP affect facility readiness adversely and may account in part for differences in IPTp3+ uptake between rounds. Multiple studies^23^^,^^31^^,^^33^ in Tanzania and other sub-Saharan African countries reported a negative association between SP stock-outs and receipt of IPTp. The greater odds of receiving IPTp3+ at hospitals than dispensaries or health centers in our study may be because SP is less subject to stock-outs in hospitals than smaller facilities. Health management information system data from the same years as the records included in this assessment revealed that 37% and 27% of MSDQI facilities in 2020 and 2021, respectively, experienced SP stock-outs (defined in Tanzania as commodity unavailability on one or more days in a month).^47^ Stock-outs were rare at hospitals (3% in 2020 and 0% in 2021), but occasional in health centers (13% in 2020 and 15% in 2021) and common in dispensaries (84% in 2020 and 85% in 2021). Stock-outs at dispensaries may affect adversely the perceptions of ANC quality provided there and may encourage “bypassing” them in favor of higher level facilities. Alternatively, pregnant women who seek ANC from hospitals may be consulting providers who have greater levels of training or more experience. In SPAs from Kenya and Namibia, ANC received from more experienced providers was associated with a greater number of ANC visits.^26^

Promoting greater number of ANC visits can increase IPTp3+ uptake.^5^^,^^48^ In 2016, the WHO shifted its focus from ANC coverage to content, as achieving the recommended number of ANC visits does not ensure receipt of recommended interventions.^11^ However, a greater number of visits was clearly associated with increased odds of receiving IPTp3+, as they offered more opportunities to obtain SP. The WHO recommends at least eight ANC contacts, which few pregnant women in our sample or other studies achieved; attendance decreases markedly in most populations after the second visit, and only 65% of Tanzanian women achieve four or more visits—more than our sample median.^49^ A lack of provider knowledge about IPTp protocols and lack of client understanding of IPTp may contribute to poor uptake and adherence.

Results from our study can guide program and policy efforts to promote ANC attendance in Tanzania and to ensure adequate stocks of SP at the dispensary level. Efforts by health-care providers and community service organizations are needed to encourage women, particularly those living in areas of high malaria transmission, to attend a sufficient number of visits and initiate ANC early enough to receive IPTp3+.^45^ Additional efforts to promote ANC and IPTp uptake, such as promotion of ANC services by community health workers or delivery of IPTp at the community level, may lead to increases in IPTp3+ coverage.^50^

### Using Malaria Services and Data Quality Improvement data to identify service delivery quality gaps.

Analysis of MSDQI data also revealed important gaps in quality of care. Coverage analysis identified hemoglobin testing as a potentially overlooked intervention during ANC, as results were not documented (i.e., not performed) for almost half of pregnant women, especially those attending ANC at dispensaries. Symptomatic MiP is a key contributor to maternal anemia, the leading cause of malaria-related morbidity and mortality among pregnant women.^2^ In Cameroon, malaria diagnosed at the first ANC visit was associated with low hemoglobin levels and anemia.^51^ In areas of high malaria transmission in Tanzania, anemia prevalence is also high. In southeastern Tanzania, a recent study^52^ reported a 68.5% anemia prevalence at the first ANC visit. Insufficient equipment and commodity availability or lack of provider skill may be responsible, as odds of anemia testing were greatest in hospitals. A study in Tanzania found that half of rural health clinics had no instrument to measure hemoglobin, and only 37% of women had received a hemoglobin test.^53^ In areas with deficient infrastructure, supply chain interventions could prevent stock-outs and improve testing rates. A cluster-randomized trial in Mozambique^54^ that distributed anemia testing supply kits with 1-day training reported that anemia screening increased from 15% at baseline to 98% during the trial. Failure to screen for anemia is a hallmark of substandard ANC. Data from Ghana and Tanzania on ANC care quality revealed that lower quality care was associated with a greater prevalence of anemia and worse maternal and perinatal outcomes.^17^ Possibly because they are usually less busy, dispensaries were more likely than health centers and hospitals to document gestational age and distribute ITNs.

### Limitations.

Our study’s retrospective design offers a descriptive portrait of MiP service provision over a fairly narrow time frame. Continued analysis of MSDQI data over time can demonstrate how MSDQI supportive supervision affects quality of care, and how these data can be used to improve data collection and documentation continuously. Working with secondary data from public-sector facilities limited the types of possible analyses. We assessed service provision as documented in ANC registers. Some MiP care components may have been provided but not documented or, less likely, recorded erroneously but not provided. Without observation to confirm receipt of these services, validity of ANC records cannot be confirmed. In addition, because ANC records did not contain screening and test results, we were unable to assess whether women received care components correctly, at the right time and frequency, and with an appropriate response.^11^ For example, we cannot ascertain whether clients with positive mRDT results were prescribed artemisinin-based combination therapy. Similarly, registers did not capture client eligibility for IPTp doses. In addition, MiP services provided by the private sector are not captured; facility readiness to provide ANC and MiP care components is generally lower in private facilities in Tanzania.^31^

Because data were collected during routine supervision, sampling was not as rigorous as in a standalone study. The MSDQI tool sampled the same number of records at all facilities regardless of client load, so our service provision analyses may overrepresent low-volume facilities. Guidelines for MiP call for the same ANC services to be provided at every facility regardless of type; oversampling is unlikely to distort care component coverage estimates. In the client satisfaction analysis, high-volume facilities are likely overrepresented because facilities without clients during supervision were omitted. Low-volume facilities may differ from high-volume facilities in client satisfaction. Future analyses could either sample facilities based on representative shares of clients by facility type and volume of clients, or use weighted analyses. In addition, our study was conducted in just three regions of Tanzania in a fraction of facilities where MSDQI is being implemented. Twenty-three other regions where MSDQI is used do not have implementation support, limiting generalizability of the findings to all of Tanzania.

The logistic regression was based on aggregated service provision data without client identifiers, so stratification by sociodemographic variables of clients was not possible. Some unmeasured demand-side factors such as concealment of pregnancy, opportunity costs, and perceptions about quality or content of care may influence timing of the first ANC visit and, consequently, total doses of IPTp pre-delivery. In addition, women may seek ANC from multiple facilities. Migration into or out of a facility catchment area during pregnancy, which is common in Tanzania, may result in incomplete records of all ANC services a particular client receives.

Last, the MSDQI client satisfaction tool relies primarily on client self-reporting, so social desirability bias could inflate satisfaction estimates. In addition, the MSDQI tool does not assess all dimensions of quality of care, including facility conditions, assurance of privacy, respectful treatment, and cost, which can influence demand for and uptake of services. These would be useful to include in MSDQI, particularly in efforts to increase uptake of ANC and IPTp services.

### Recommendations for quality malaria in pregnancy service delivery.

In Tanzania, achieving quality improvement for MiP requires attention be paid to ANC provision beyond mere coverage.^55^ Multiple strategies to increase IPTp uptake and quality of documentation are underway. Registers can now record up to four doses of IPTp, preservice MiP training curriculum are strengthening provider skills, and IPTp3+ and SP availability are included among indicators used to determine cash incentives for providers and facilities under results-based financing schemes.^9^ Implementing partners have promoted IPTp and early and continued ANC attendance, stock status monitoring for MiP commodities, and periodic data review and quality assessment meetings at facilities. These are critical inputs to improve health system readiness to provide quality MiP services.^13^

No single tool, method, or indicator can capture all dimensions of quality of care (e.g., facility readiness, provider performance, number of visits, timing of ANC initiation, coverage, and client satisfaction).^18^ Using multiple data sources can paint a more detailed picture of both MiP care component coverage and quality, and identify issues affecting service delivery (e.g., stock-outs, long wait times, and late initiation of ANC). The MSDQI tool—and OTSS tools for MiP used in other countries—could be adapted to capture more effectively the appropriateness of care provision and other dimensions of quality of care, such as respectful maternity care and costs. Routinizing collection of data that capture both content and experience of MiP services through modalities such as supportive supervision is needed to mark progress toward quality improvement.

## CONCLUSION

Our study provides insight on the quality of MiP services delivered during ANC visits in Tanzania, demonstrating how routinely collected data can support quality improvement by identifying gaps in service delivery and guiding quality improvement initiatives. Quality of MiP services can be evaluated through several different lenses, including service provision and client experiences of care. Evaluating how supportive supervision approaches affect health service performance helps strengthen the case for MOHs to adopt supportive supervision approaches using tools such as the MSDQI tool to collect data that are useful for monitoring coverage and quality of MiP services.

## Supplemental Materials

10.4269/ajtmh.23-0399 Supplemental Materials
