# Supplementary material for: Understanding Antenatal Care Service Quality for Malaria in Pregnancy through Supportive Supervision Data in Tanzania
Source: Am J Trop Med Hyg. 2024 Feb 6;110(3 Suppl):56–65. doi: 10.4269/ajtmh.23-0399 (PMC10919228; doi:10.4269/ajtmh.23-0399)
Supplement: Supplemental Materials [file tpmd230399.SD1.pdf]

Table S1. Client sampling for client satisfaction survey

		Round 1 (2021)	Round 2 (2022)	Total Clients Surveyed
0 clients	Dispensary	198	212	0
	Health Center	21	15	0
	Hospital	6	2	0
	**Total**	**225**	**229**	**0**
1 client	Dispensary	55	56	111
	Health Center	10	14	24
	Hospital	0	2	1
	**Total**	**65**	**72**	**137**
2 clients	Dispensary	164	170	668
	Health Center	30	15	90
	Hospital	8	5	26
	**Total**	**202**	**190**	**784**
3 clients	Dispensary	0	1	3
	Health Center	0	8	24
	Hospital	0	1	3
	**Total**	**0**	**10**	**30**
4 clients	Dispensary	0	1	4
	Health Center	0	14	56
	Hospital	0	6	24
	**Total**	**0**	**21**	**84**
Totals	**Total discrete facilities**	**492**	**522**	
	**Total facilities with clients**	**267**	**293**	
	**Total clients surveyed**	**469**	**566**	**1035**

Table S2. Association of health facility type with coverage of Malaria in Pregnancy care components (regression results)

**Health facility type**	**Malaria in Pregnancy Care Component Coverage*** OR [CI] *p*-value						
	**ITN provision**	**Hb test recorded**	**mRDT result**	**Hematinic provision**	**GA recorded**	**Any IPTp**	**IPTp3+**
Dispensary	REF	REF	REF	REF	REF	REF	REF
Health Center	**0.43 [0.32-0.57] p<0.001**	**1.24 [1.11-1.30] p<0.001**	0.87 [0.67-1.10] p=0.27	1.03 [0.83-1.27] p=0.79	**0.80 [0.64-0.99] p=0.04**	**1.16 [1.01-1.32] p=0.03**	0.90 [0.80-1.00] p=0.06
Hospital	0.82 [043-1.57] p=0.15	**1.54 [1.25-1.90] p<0.001**	0.72 [0.43-1.10] p=0.20	0.74 [0.53-1.05] p=0.08	**0.64 [0.44-0.91] p=0.01**	0.82 [0.63-1.06] p=0.13	**1.34 [1.08-1.67] p=0.007**

Regression analyses include all client records where facility was specified (N=11,256). ITN: insecticide-treated net; Hb: hemoglobin; mRDT: malaria rapid diagnostic test; GA: gestational age; IPTp: intermittent preventive treatment in pregnancy

Table S3. Association of health facility type with receipt of total number of IPTp doses (regression results)

Health facility type	Total number of IPTp doses received* OR [CI] *p*-value		
	**1**	**2**	**3+**
Dispensary	REF	REF	REF
Health Center	**1.16 [1.02-1.33] p=0.025**	1.0 [0.87-1.15] p=0.99	0.90 [0.80-1.00] p=0.059
Hospital	0.82 [0.63-1.07] p=0.15	0.78 [0.59-1.02] p=0.07	**1.34 [1.08-1.67] p=0.007**
Unspecified	**0.11 [0.02-0.84] p=0.033**	N/A**	**23.13 [3.16-169.52] p=0.002**

** Each of the 3 regressions in this table was performed on the subset of women documented as receiving that total number of doses of IPTp. Women who received zero doses (N=2,291) were not included. IPTp: intermittent preventive treatment in pregnancy; **Could not be computed due to small sample size*
